# Pediatric patient with maturity-onset diabetes of the young type 5 and 17q12 deletion syndrome: A case report

**DOI:** 10.3892/mi.2025.260

**Published:** 2025-08-11

**Authors:** Marta Rico-Rodríguez, Sandra Fuentes-Cantero, Marta Carolina García-Rivera, Gema María Varo-Sánchez

**Affiliations:** Laboratory Medicine Department, Riotinto Hospital (Huelva North Health Management Area), 21660 Minas de Riotinto, Huelva, Spain

**Keywords:** diabetes mellitus, maturity-onset diabetes of the young type 5, hyperglycemia, deletion syndrome, pediatric endocrinology

## Abstract

Maturity-onset diabetes of the young type 5 (MODY5), a rare disease, is often misdiagnosed as type 2 diabetes, leading to delays in diagnosis. 17q12 deletion syndrome is a rare chromosomal abnormality, the signs and symptoms of which can vary widely among different patients, including those with MODY5. The present study reports the case of a 9-year-old patient with chromosomal 17q12 deletion syndrome identified by the *de novo* deletion of the 1.49 Mb segment in the 17q12 band of the HNF1B gene by genetic analysis. The patient presented with MODY5, a short stature and hypertransaminasemia as clinical features. The risk of inheritance of 17q12 deletion syndrome is ~50%, and genetic counseling is recommended for all patients who are suspected to have or are diagnosed with 17q12 deletion syndrome.

## Introduction

Maturity-onset diabetes of the young (MODY) is the most frequent form of monogenic diabetes and is characterized by presenting autosomal-dominant inheritance, discrete hyperglycemias, the absence of pancreatic β-cell autoimmunity with conserved C-peptide levels and an early onset, usually prior to the age of 25 years (1). The clinical features and treatment of this disease depend on the specific variant affected, which involves personalized therapeutic approaches.

The prevalence of MODY is estimated to be between ~1.1 and 6.5% of the pediatric diabetic population, with a high degree of geographic variability. The GCK-MODY, HNF1A-MODY and HNF4A-MODY forms account for >80% of MODY cases (2). Of note, 50-90% of MODY cases are misdiagnosed as type 1 or 2 diabetes (3).

The present study reports a new case of MODY5 as a feature of 17q12 deletion syndrome. 17q12 deletion syndrome usually includes MODY5, structural or functional abnormalities of the kidneys, and neurodevelopmental or neuropsychiatric disorders.

## Case report

A 9-year-old male patient was referred from primary care to the Pediatric Endocrinology Department for hyperglycemia under study at the Riotinto Hospital (Minas de Riotinto, Spain); he was also being followed-up for a short stature.

He was admitted to the referral hospital on June 20, 2022 for glycemic profiling and to complete the analytical study. The patient remained asymptomatic during his admission. Feeding and diuresis were normal. Analytical control was performed during his stay, and the fasting blood glucose level was 121 mg/dl. The hemogram was normal and the test for celiac disease yielded negative results. With respect to the autoimmunity analysis, all parameters were negative. The results of the analysis are presented in Table I.

The patient was discharged with a diagnosis of isolated hyperglycemia, without diagnostic criteria for diabetes mellitus (DM) or MODY, as well as hypertransaminasemia, and increased serum ketone levels. An analysis of persistent hypertransaminasemia was performed; a complete liver analysis with a biopsy yielded normal findings, with no relevant histological alterations in reported in the liver parenchyma.

As the patient continued with impaired fasting blood glucose levels, it was thus decided to perform a genetic analysis to search for variants in genes related to MODY using massive next-generation sequencing (NGS), which was performed by an external laboratory. This is a customized panel for analyzing genes associated with diabetes mellitus. A panel including 14 MODY-related genes (GCK, HNF1A, HNF4A, HNF1B, ABCC8, KCNJ11, INS, NEURD1, CEL, APPL1, PDX1, KLF11, PAX4 and BLK) Capture is based on WES. The kit used is the Twist Bioscience for Illumina Exome 2.5 Panel (ref. 20077596). The list of genes can be found in the report. Extraction kit: Promega Maxwell RSC Whole Blood DNA kit; cat. no. AS1520, Promega Corporation. Bioanalyzer: Agilent Tape Station 4200. 100 bp paired end. Illumina. NovaSeq 6000 S4 Rgt Kit v.1.5 (200 cyc.); cat. no. 20028313, Agilent Technologies, Inc. Software: DRAGEN Enrichment, version 3.8.4. RefLab Interpreter (in-house developed software). The raw DNA-Seq sequencing data were uploaded to NCBI with Bioproject ID: 1279590.

The genetic analysis identified the presence in heterozygosis of a pathogenic CNV (copy number variation) deletion of ~1.49 Mb in chromosomal region 17q12, comprising the HNF1B gene included in the study. As result the patient presented pathogenic mutation associated with MODY5, this result being compatible with the phenotype described in the patient.

As an action strategy, it was recommended that a sensor be attached to the patient for 15 days to evaluate the glycemia pattern throughout the day, as well as the avoidance of complex absorption carbohydrates and refined sugars, and to insist on physical exercise. The next control will be performed in 4 months with the determination of HbA1c levels.

Sensor data reported stable blood glucose levels and HbA1c at 5.5%; therefore, it was decided to delay the initiation of insulin therapy.

## Discussion

The monogenic MODY phenotype is associated with certain variants in multiple genes. The identification of these genetic variants drives different therapeutic strategies, in addition to microvascular and macrovascular complications (4). The patient with MODY5 reported herein presented the 17q12 deletion included in the HNF1B gene. 17q12 deletion syndrome is a rare chromosomal anomaly with an estimated prevalence of 1.6 per 100,000 citizens, and high penetrance and variable expressivity (5).

The majority of these genes involved encode transcription factors expressed in β-cells of the pancreas (1). MODY subtype 3 is the most common form of MODY, resulting in familial symptomatic diabetes. It is caused by a heterozygous pathogenic variant in HNF1A, which encodes a transcription factor (hepatocyte nuclear factor 1A) that is critical for pancreatic differentiation and function (6), whereas the MODY5 subtype present in the patient described herein is caused by a 17q12 deletion included in the HNF1B gene. This variant affects transcription factor HNF1B, which is involved in the organogenesis of the kidney, genitourinary tract, liver, lungs, intestine and pancreas (7); hence it is also known as renal cyst and diabetes (RCAD) syndrome (8).

The main presentation includes renal cysts and/or dysplasia, followed by diabetes during adolescence or early adulthood. In addition, the clinical spectrum may include pancreatic hypoplasia, exocrine pancreas deficiency and genitourinary abnormalities (9). In patients with DM, the presence of cystic kidneys and elevated levels of liver enzymes can be used as predictors of an HNF1B mutation (10). Although MODY5 develops in early adulthood, carriers of HNF1B mutations have significantly reduced birth weights (11), as was the case with the patient in the present study. *De novo* genetic variants or deletions account for one- to two-thirds of cases and, thus, there may be no family history of diabetes in first-degree relatives (12). Insulin is the first-line treatment option for this type of MODY since, unlike the MODY1 form, patients with MODY5 do not respond to oral antidiabetic treatments, such as sulfonylureas (13). Renal management is a particularly critical aspect of treatment in patients carrying HNF1B variants, as these individuals will develop renal dysfunction by the age of 45 years, and half will progress to end-stage renal disease (13,14).

In the case described in the present study, the increase in glucose and transaminase levels, in conjunction with the other tests performed, supports the performance of screening tests for HNF-1B mutations; according to the literature, genetic testing for HNF-1B mutations is justified in non-obese diabetic subjects with slowly progressive non-diabetic nephropathy, particularly when there are renal or genital malformations or functional liver disorders, regardless of a family history of diabetes (15). With reference to the increase in ketones found in the analytical study, this is not related to MODY, but could be secondary to the pre-analytical conditions of the patient, such as prolonged fasting or admission stress.

The development of more advanced molecular techniques, such as NGS, has had a major impact on the identification of variants associated with MODY. Several gene panels are available for diagnosis (16). In addition, all patients with suspected or diagnosed 17q12 deletion syndrome should be offered genetic counseling as it presents a 50% risk of inheritance (17).

In conclusion, although there is ample clinical evidence suggesting the diagnosis of this type of diabetes, as discrete hyperglycemias, the absence of pancreatic β-cell autoimmunity with conserved C-peptide levels and an early onset, there is no single clinical criterion. Genetic diagnosis and proper clinical evaluation of the different MODY forms has a significant effect on the lives of these patients, as it leads to the interruption and/or modification of unnecessary treatments and hinders progress towards personalized medicine.

## Figures and Tables

**Table I:** Summary of the analytical results of the patient.

Test/parameter	Results	Reference values	Units
Glucose	121^a^	60-100	mg/dl
Glycosylated hemoglobin (HbA1c)	5.5	<6.5	%
Insulin	10.24	2.6-24.90	µUI/ml
C-peptide	2.5	1.1 4.4	ng/ml
Ketones	8.9^a^	0.2-2.8	mg/dl
Aspartate transaminase	72.5^a^	0-48	U/l
Alanine transaminase	74.3^a^	5-39	U/l
Insulinoma associated autoantibodies (IA-2)	0.22	0-28	UI/ml
Glutamic descarboxylase 65 kD (GAD-65)	<0.10	0-17	UI/ml
Insulin autoantibodies (IAA)	4.6	<8.2	%
Islet cell autoantibodies (ICA)	<1/2	<1/2	-

^a^Results outside the normal range.

## Data Availability

The raw DNA-Seq sequencing data generated in the present study may be found in NCBI with the Bioproject ID: PRJNA1279590 (http://ncbi.nlm.nih.gov/bioproject/1279590).
